# Cleavage of Type I Collagen by Fibroblast Activation Protein-α Enhances Class A Scavenger Receptor Mediated Macrophage Adhesion

**DOI:** 10.1371/journal.pone.0150287

**Published:** 2016-03-02

**Authors:** Anna Mazur, Emily Holthoff, Shanthi Vadali, Thomas Kelly, Steven R. Post

**Affiliations:** 1 Interdisciplinary Biomedical Sciences Program, University of Arkansas for Medical Sciences, Little Rock, Arkansas, United States of America; 2 Department of Pharmacology and Toxicology, University of Arkansas for Medical Sciences, Little Rock, Arkansas, United States of America; 3 Department of Pathology, University of Arkansas for Medical Sciences, Little Rock, Arkansas, United States of America; University of California, San Diego, UNITED STATES

## Abstract

Pathophysiological conditions such as fibrosis, inflammation, and tumor progression are associated with modification of the extracellular matrix (ECM). These modifications create ligands that differentially interact with cells to promote responses that drive pathological processes. Within the tumor stroma, fibroblasts are activated and increase the expression of type I collagen. In addition, activated fibroblasts specifically express fibroblast activation protein-α (FAP), a post-prolyl peptidase. Although FAP reportedly cleaves type I collagen and contributes to tumor progression, the specific pathophysiologic role of FAP is not clear. In this study, the possibility that FAP-mediated cleavage of type I collagen modulates macrophage interaction with collagen was examined using macrophage adhesion assays. Our results demonstrate that FAP selectively cleaves type I collagen resulting in increased macrophage adhesion. Increased macrophage adhesion to FAP-cleaved collagen was not affected by inhibiting integrin-mediated interactions, but was abolished in macrophages lacking the class A scavenger receptor (SR-A/CD204). Further, SR-A expressing macrophages localize with activated fibroblasts in breast tumors of MMTV-PyMT mice. Together, these results demonstrate that FAP-cleaved collagen is a substrate for SR-A-dependent macrophage adhesion, and suggest that by modifying the ECM, FAP plays a novel role in mediating communication between activated fibroblasts and macrophages.

## Introduction

Solid epithelial tumors evoke a reactive stromal response that is critical for growth and progression of the tumor. A reactive stroma is complex and consists of activated fibroblasts, newly formed vasculature, infiltrating immune cells, and extracellular matrix (ECM). Soluble signaling molecules such as cytokines and growth factors are well-documented mediators of interactions between cells in the tumor microenvironment. The tumor ECM also mediates communication between various cell types, in part by providing migration and adhesion signals [1]. Although increased deposition and cross-linking of collagen in the tumor stroma is associated with increased tumor growth [1–4], little is known about how specific ECM adhesion signals are created and regulated. It has been suggested that proteolytic cleavage of collagen increases cancer growth, invasion, and angiogenesis [5, 6]. Thus is likely that signals induced by changes in the ECM act in conjunction with soluble cytokines produced by other stromal components to modulate tumor growth.

Tumor associated fibroblasts (TAFs) are a key element of the reactive stroma. TAFs are fibroblasts that have undergone a major phenotypic change to an activated state characterized by increased proliferation, secretion of type I collagen, and expression of ECM-degrading proteases [7]. TAF-derived proteases present in the tumor microenvironment play a pivotal role in remodeling the ECM to make it permissive for tumor cell invasion and infiltration by normal endothelial cells and immune cells such as macrophages. Fibroblast Activation Protein-α (FAP) is a membrane-bound, serine protease that is expressed by activated fibroblasts including TAFs, but is absent from normal healthy adult tissues [8–10]. Although FAP reportedly cleaves type I collagen in vitro [11–14], the pathophysiological significance of FAP-generated collagen cleavage products is unclear. One possibility is that FAP participates in modifying ECM molecules to create and regulate cell adhesion in the tumor microenvironment.

Macrophages are another major cellular component of the tumor stroma and their infiltration and accumulation in the tumor microenvironment is correlated with tumor progression, invasion, and poor patient prognosis [15–18]. Tumor associated macrophages (TAMs) typically exhibit an M2 phenotype and are associated with secretion of a wide variety of growth factors, cytokines, and chemokines that suppress an anti-tumor immune response and promote tumor growth [16, 17]. Macrophages infiltrate and are retained in the tumor microenvironment through expression of specific adhesion proteins, such as integrins and scavenger receptors, that bind and mediate the adhesion of cells to ECM components of the tumor stroma. Interestingly, macrophages do not adhere well to native type I collagen [19, 20], which is the most abundant ECM protein in a tumor stroma [21]. This suggests that modifications of collagen are necessary for macrophage adhesion. Based on this background, we speculated that FAP, which is expressed by TAFs, cleaves collagen and converts it into an adhesive substrate for macrophages.

## Materials and Methods

### Animals

Animals used in this study include C57Bl/6 mice and SRA^-/-^ mice in a C57Bl/6 background purchased from Jackson Laboratories (Bar Harbor, ME, USA); and MMTV-PyMT obtained from Dr. S. Gendler (Mayo Clinic, Scottsdale, AZ). All animals were maintained as colonies at the University of Arkansas for Medical Sciences and housed on a 12-hour light-dark cycle. To generate a breast tumor model, male MMTV-PyMT mice and C57Bl6 female mice were bred, and the offspring genotyped for the presence of MMTV-PyMT transgene. Breast tumors from female offspring that were heterozygous for MMTV-PyMT were isolated and used for tumor immunohistochemistry. All animals were provided food (Teklan Global 16% protein rodent diet; Harlan Laboratories, Indianapolis, IN, USA) and water *ad libitum*. Animal care and use were performed according to protocols reviewed and approved by the Institutional Animal Care and Use Committee at the University for Arkansas for Medical Sciences.

### Protease activity assays

Recombinant human FAP (R&D Systems, Minneapolis, MN, USA) was incubated with dye quenched (DQ) type I collagen or type IV collagen (100 μg/ml; Life Technologies, Carlsbad, CA, USA) in assay buffer according to manufacturer’s instructions. Proteolytic cleavage of each DQ substrate was assessed by measuring fluorescence in a Synergy-2 plate reader (Biotek, Winooski, VT, USA) using excitation/emission wavelengths of 485/528 nm. Baseline fluorescence was determined by incubation of DQ substrates in assay buffer in the absence of FAP. As a control for proteolytic activity, FAP was incubated with its synthetic substrate Z-Gly-Pro-AMC (Bachem, Bubendorf, Switzerland) according to manufacturer’s instructions.

### Cell isolation and treatment

Mouse peritoneal macrophages (MPMs) were isolated from C57BL/6J and SR-A^-/-^ mice via peritoneal lavage with sterile saline from non-injected mice (for spreading assays) or from mice injected intraperitoneally with 4% thioglycollate 4 days prior to isolation (for attachment assays). For each assay, qualitatively similar results were obtained in preliminary experiments using non-elicited and elicited macrophages (data not shown). Isolated cells were immediately resuspended in DMEM GlutaMax (Life Technologies, Carlsbad, CA, USA) supplemented with FBS (10% vol/vol, Atlanta Biologicals, Flowery Branch, GA, USA), and penicillin/streptomycin (1%). Cell number and viability were assessed prior to use in experiments.

### Macrophage adhesion assays

Macrophage attachment was assessed as described previously [22, 23]. Briefly, 12-well tissue culture dishes were coated for 5 h at 37°C with 10 μg/cm^2^ of type I collagen (Stem Cell Technologies, Vancouver, Canada) or fibronectin (Sigma, St. Louis, MO, USA). Collagen coated wells were then treated (24 h; 37°C) with buffer (control), recombinant FAP (1 μg/ml), or FAP that was inhibited with 4 mM PMSF or heat inactivated at 80°C for 10 min. The treated wells were washed, and freshly isolated MPMs (10^6^ cells/well) plated for 30 min at 37°C. Non-adhered cells were removed by washing with PBS, and the number of cells remaining attached were quantified using a hemocytometer.

Macrophage spreading was similarly examined [22, 23]. Tissue culture chamber slides (Nalge Nunc International, Naperville, IL, USA) were coated with type I collagen (10 μg/cm^2^; 4°C; 24 h), and then treated for 24 h at 37°C with buffer (control), active FAP, or heat inactivated FAP (1 μg/ml). Treated wells were washed, and freshly isolated MPMs (0.15 x 10^6^ cells/chamber) plated for 2 hr at 37°C. Non-adherent cells were removed, and then adherent cells were fixed with 4% paraformaldehyde and permeabilized with 0.1% Triton X-100. Cells were stained with Alexa-Fluor 647-conjugated phalloidin (Life Technologies) and nuclei were stained with DAPI. Images (40x) were digitally captured with Olympus CKX41 microscope and the surface area of cells quantified using AxioVision software (Carl Zeiss, Jena, Germany). Five independent images with at least 75 cells total were quantified in each experiment.

### Immunohistochemistry of MMTV-PyMT tumors

Tumor tissues were isolated from MMTV-PyMT females before individual tumors reached 1 cm in any dimension. Isolated tumors were immediately embedded in OCT medium, rapidly frozen in liquid nitrogen, and stored at -80°C. Sections (8 μm) were cut onto glass slides and tissues fixed by incubation in ice-cold acetone for 20 min. Endogenous peroxidases were blocked with a dual endogenous enzyme block (Dako North America, Carpinteria, CA, USA). Additional blocking was performed with serum-free protein block (Dako) and 2.5% horse serum (Vector, Burlingame, CA, USA). To detect collagen, tissues were incubated with an anti-collagen antibody (Rabbit Anti-Mouse, EMD Millipore, Billerica, MA, USA), followed by Vector ImmPress Anti-Rabbit Reagent for alkaline phosphatase, and Vector ImmPact Red Substrate Solution. A dual-staining approach was used to identify SR-A-expressing macrophages and FAP-expressing fibroblasts. Following the blocking steps outlined above, tissues were incubated with primary anti-SR-A antibody (Goat Anti-Mouse, R&D Systems), then treated with Vector ImmPress Anti-Goat Reagent for peroxidase and Vector Immpact DAB Solution to visualize SR-A staining in brown. A second blocking step was then performed with 2.5% horse serum, and tissue sections incubated with primary anti-FAP antibody (Rabbit Anti-Mouse, Millipore), followed by Vector Immpress Anti-Rabbit Reagent for alkaline phosphatase and Vector Immpact Red Substrate Solution to visualize FAP staining in red. Tumor sections were counterstained with hematoxylin, dehydrated and coverslipped.

### Statistical analysis

As indicated in individual figure legends, experiments were repeated at least three times and data analyzed with GraphPad Prism software using a *t*-test for comparing 2 groups, or ANOVA followed by the appropriate post-hoc statistical test to compare multiple groups. Differences with *p* < 0.05 were considered statistically significant.

## Results

### FAP selectively cleaves type I collagen

**Fig 1:**
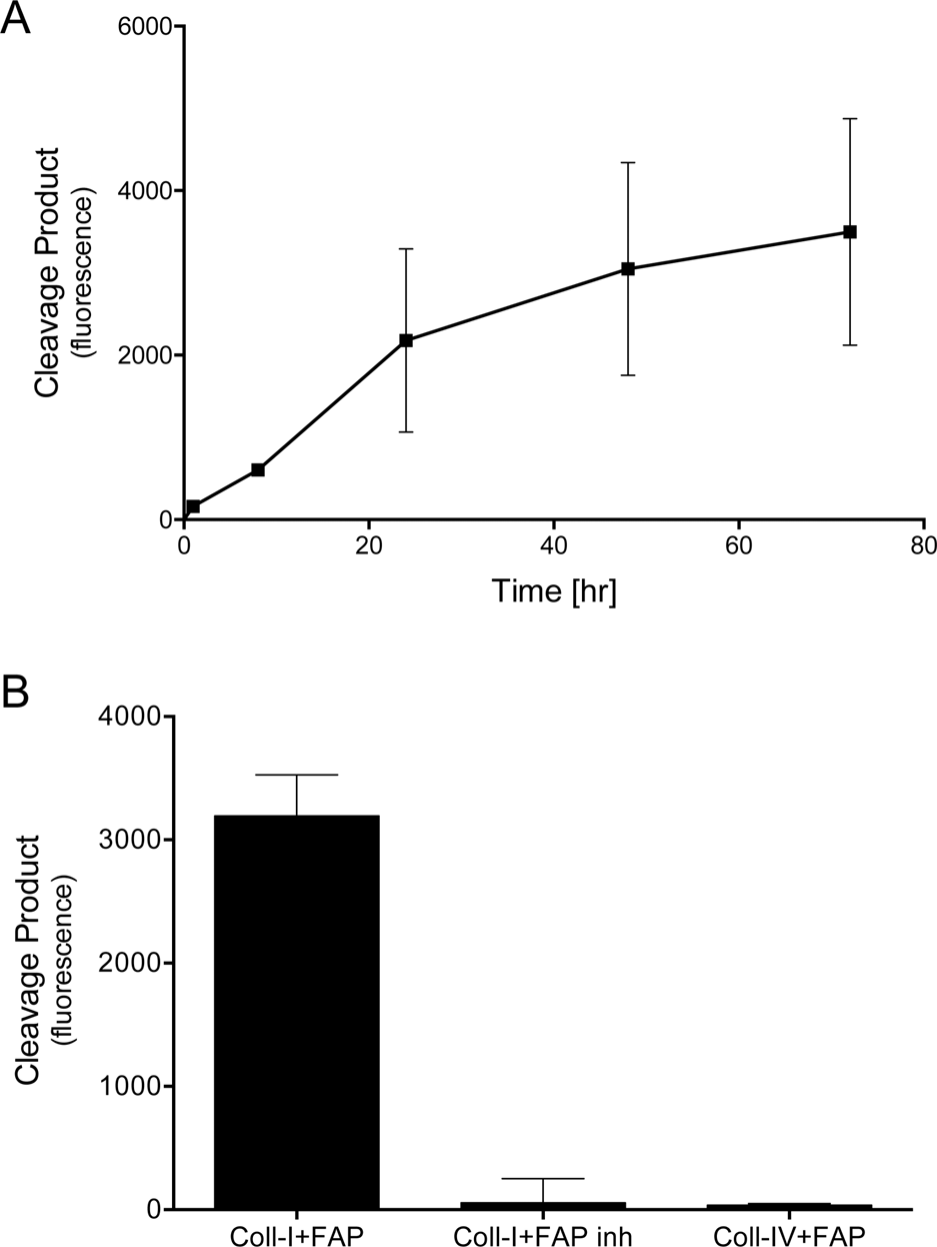
FAP selectively cleaves type I collagen. (A) DQ type I collagen was incubated with FAP (1 μg/ml) for increasing times and the extent of substrate cleavage quantified by measurement of increasing fluorescence. (B) DQ type I collagen was incubated with active or PMSF inhibited FAP (1 μg/ml) and DQ type IV collagen was incubated with active FAP at the same concentration for 24 hr. Degradation of DQ collagens was quantified by measurement increasing fluorescence. Shown are the means ± SD of at least 3 experiments.

FAP has been reported to cleave type I collagen [11–14], which is the most abundant ECM protein in a tumor stroma [21]. This activity was confirmed using recombinant FAP and fluorescently quenched (DQ) collagen substrates that fluoresce when cleaved. As shown in Fig 1, incubating FAP (1 μg/ml) with DQ type I collagen resulted in a time-dependent increase in fluorescence that was most evident during the first 24 h (Fig 1A). Inhibiting FAP proteolytic activity with PMSF (Fig 1B) or by heat-inactivation (data not shown) prior to incubation with DQ type I collagen abolished the FAP dependent increase in fluorescence. In contrast to its effect on type I collagen, there was no increase in fluorescence when FAP was incubated with DQ type IV collagen (Fig 1B). Together these results demonstrate that FAP selectively cleaves type I collagen, and establish conditions (1 μg/ml, 24 h) that were used to examine the consequence of FAP-mediated collagen cleavage in the macrophage adhesion assays described below.

### FAP-mediated cleavage of type I collagen increases macrophage adhesion

**Fig 2:**
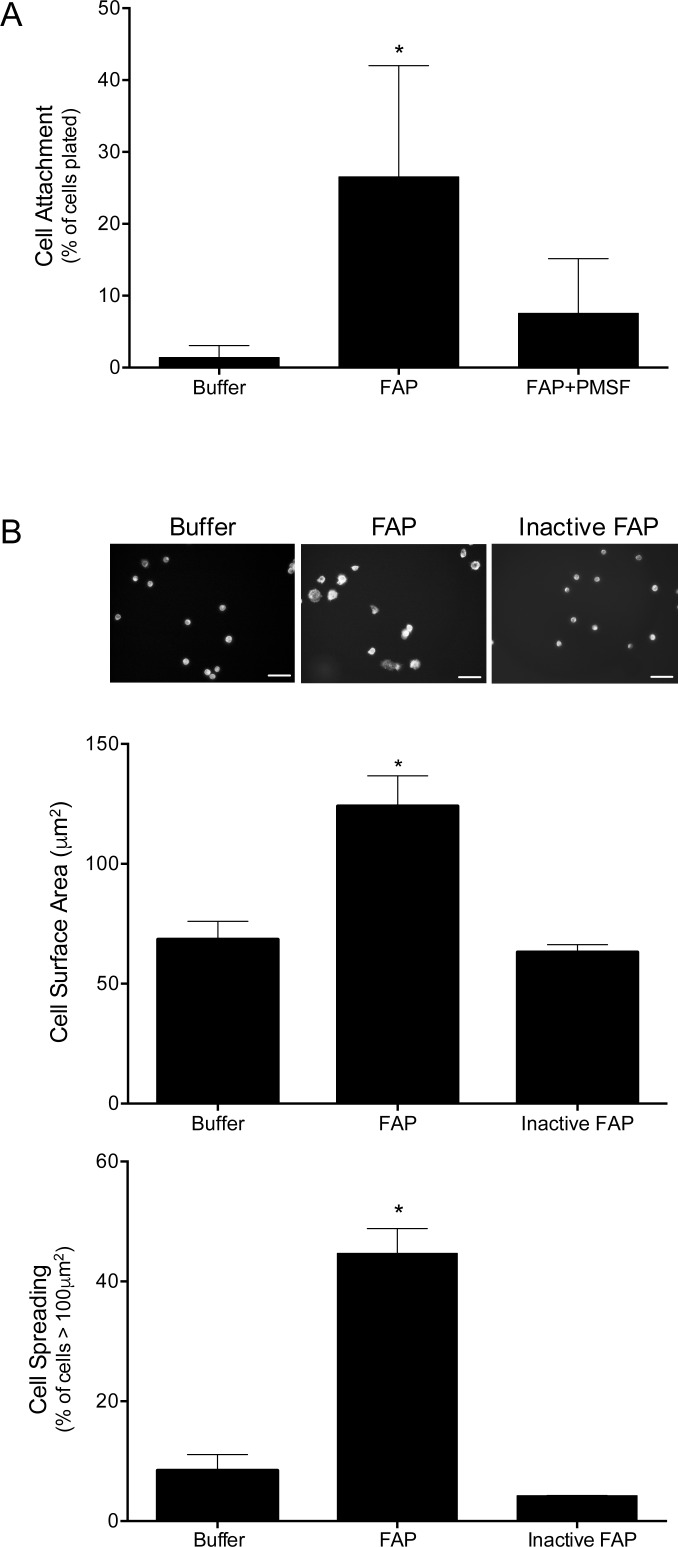
FAP-mediated cleavage of type I collagen enhances macrophage adhesion. (A) MPMs were adhered to type I collagen that was pretreated with buffer, FAP, and FAP inhibited with PMSF. Non-adhered cells were removed by washing, and the number of attached cells quantified and expressed as a percentage of total cells plated. Shown are the means ± SD of 4 experiments. Results were compared by one-way ANOVA with Tukey’s post-hoc test. (B) MPMs were adhered to type I collagen pretreated with buffer or FAP. Non-adhered cells were removed by washing, and attached cells were fixed and stained with fluorescent phalloidin and DAPI. Representative images were digitally captured and the surface area of cells quantified. Scale bars = 30 μm. Shown are the means ± SD of cell surface area and percentage of cells displaying a surface area > 100 μm^2^ from 3 experiments. Results were log-transformed and compared by one-way ANOVA with Dunnett’s post-hoc test. * indicates significant (p<0.05) difference from cells plated on buffer treated collagen.

Specific modifications of collagen have been shown to enhance macrophage adhesion [19, 20, 24, 25]. We therefore tested whether macrophage adhesion to type I collagen was increased following FAP-mediated cleavage (Fig 2). Primary MPMs were plated for 30 min (to assess attachment) or 2 h (to assess spreading) on type I collagen-coated tissue culture dishes that were untreated, treated with catalytically active FAP, or treated with FAP that was inhibited with PMSF (FAP + PMSF) or heat inactivation (inactive FAP). Macrophages attached poorly (≤ 1% of plated cells) to untreated (native) type I collagen (Fig 2A). In contrast, the number of attached macrophages was significantly increased (> 30% of plated cells) when plated on collagen treated with active FAP, but not when plated on collagen treated with PMSF-inhibited FAP. Similarly, relative to macrophages plated for 2 h on native type I collagen, macrophages that were plated on type I collagen that was treated with active FAP exhibited enhanced spreading as evidenced by a significant increase in surface area with a high percentage of cells having a surface area > 100 μm^2^ (Fig 2B). Macrophage spreading on collagen treated with inactive FAP was similar to that on native collagen. Overall, these results demonstrate that macrophage adhesion to type I collagen is substantially enhanced by FAP-mediated cleavage.

### Macrophage adhesion to FAP-cleaved collagen is integrin-independent

**Fig 3:**
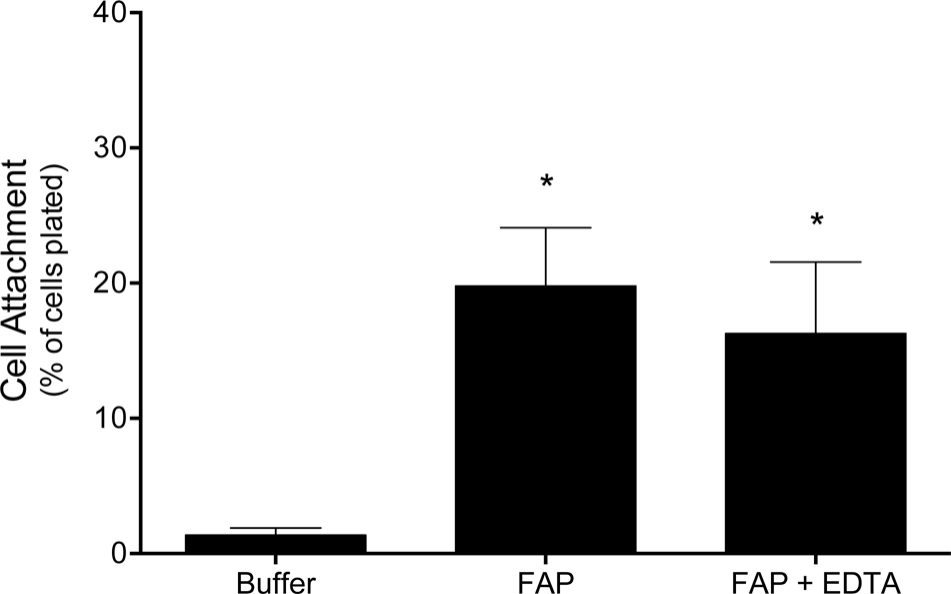
Macrophage adhesion to FAP modified collagen is integrin independent. Type I collagen-coated plates were pretreated with buffer or FAP, and then MPMs were adhered in the presence or absence of EDTA. Non-adhered cells were removed by washing, and the number of remaining attached cells quantified and expressed as a percentage of total cells plated. Shown are the means ± SD of 4 experiments. Results were compared with one-way ANOVA with Tukey’s post hoc test. * indicates significant (p<0.05) difference from cells plated on buffer treated collagen.

Integrins are widely expressed surface proteins that mediate Ca^2+^-dependent cell adhesion to ECM, thus sequestering extracellular cations can disrupt this interaction [26–29]. To determine whether increased macrophage adhesion to FAP-cleaved type I collagen is mediated by integrins, macrophage attachment was examined following chelation of divalent cations with EDTA. Primary MPMs were adhered to untreated (control) and FAP-cleaved type I collagen for 30 min in the presence or absence of EDTA (5 mM). Results depicted in Fig 3 show that macrophage attachment to type I collagen was substantially increased by FAP-mediated cleavage, and this increased attachment was not impaired by EDTA treatment. To confirm that integrin-mediated adhesion could be inhibited with this approach, MPM adhesion to fibronectin was tested in the presence and absence of EDTA. Over 80% of plated macrophages adhered to fibronectin in the absence of EDTA; however <10% adhered in the presence of EDTA confirming disruption of integrin-mediated adhesion (data not shown). The divalent cation independence of the increased macrophage adhesion to FAP-cleaved type I collagen indicates that this adhesion is not mediated by integrins.

### Macrophage adhesion to FAP-cleaved collagen is mediated by SR-A

**Fig 4:**
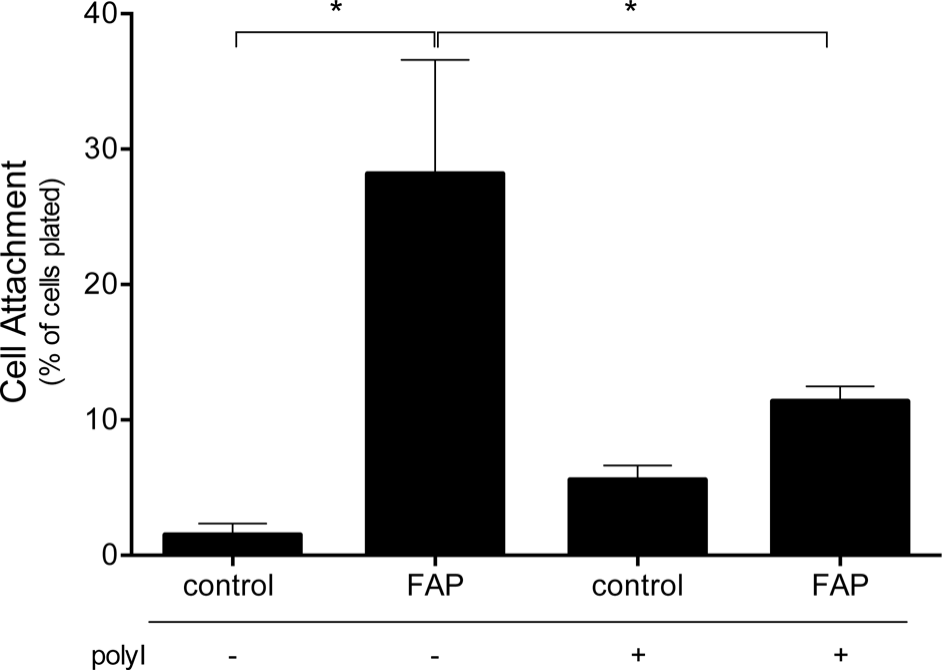
Polyinosine inhibits macrophage adhesion to FAP-modified type I collagen. (A) Type I collagen-coated plates were pretreated with buffer or FAP, and then MPMs were adhered in the presence or absence of polyinosine. Non-adhered cells were removed by washing, and the number of attached cells quantified and expressed as a percentage of total cells plated. Shown are the means ± SD of 4 experiments. Results were compared by *t*-test. * indicates significant (p<0.05) differences between groups.

Macrophages express a variety of receptors that recognize modified ECM components. In particular, SR-A mediates the divalent cation independent macrophage adhesion to a variety of substrates including glucose-modified collagen, denatured collagen, and collagenase-cleaved type I collagen [20, 25, 30]. Therefore, we determined whether SR-A mediates macrophage adhesion to FAP-cleaved type I collagen. MPMs were adhered to tissue culture plates that were coated with type I collagen and left untreated (control) or treated with active FAP in the presence or absence of polyinosine, which is used to antagonize SR-A binding [31]. As shown in Fig 4, increased adhesion of MPMs to FAP-cleaved collagen was significantly inhibited in the presence of polyinosine. This result is consistent with a role for SR-A in mediating macrophage adhesion to FAP-cleaved collagen.

**Fig 5:**
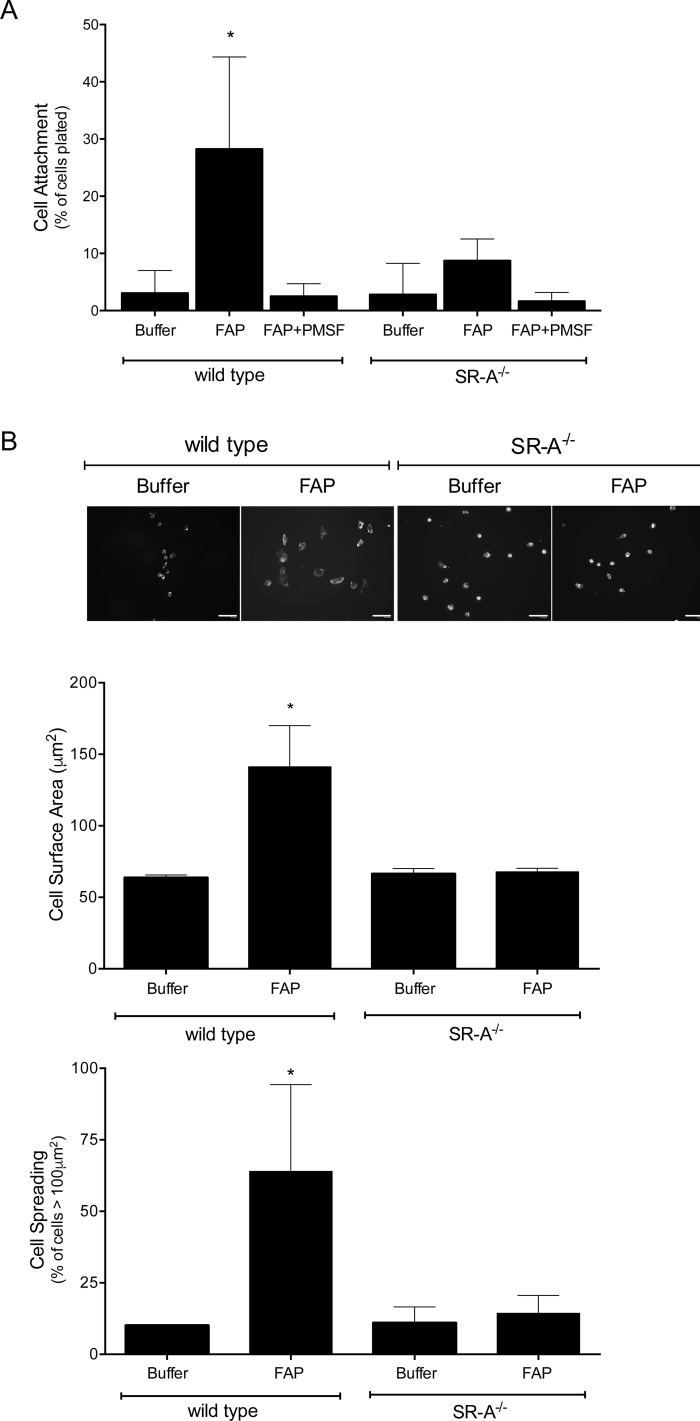
SR-A mediates macrophage adhesion to FAP-modified type I collagen. (A) MPMs isolated from wild-type (SR-A^+/+^) or SR-A^-/-^ mice were adhered to type I collagen pretreated with buffer, FAP, or PMSF-inhibited FAP. Non-adherent cells were removed by washing, and the number of attached cells quantified and expressed as a percentage of total cells plated. Shown are the means ± SD of 4 experiments. Results were compared by one-way ANOVA with Tukey’s post-hoc test. (B) MPMs were adhered to type I collagen pretreated with buffer or FAP. Non-adhered cells were removed by washing, and attached cells were fixed and stained with fluorescent phalloidin and DAPI. Representative images were digitally captured and the surface area of cells quantified. Scale bars = 30 μm. Shown are the means ± SD of cell surface area and percentage of cells displaying a surface area > 100 μm^2^ from 3 experiments. Results were compared by *t*-test. * indicates significant (p<0.05) difference from wild-type macrophages plated on buffer treated collagen.

Although polyinosine binds SR-A, it is not specific for SR-A. Therefore, we compared macrophage adhesion to FAP-cleaved type I collagen using MPMs isolated from wild type (SR-A^+/+^) and SR-A knock out (SR-A^-/-^) mice. Primary MPMs were plated for 30 min (to assess attachment) or 2 h (to assess spreading) on type I collagen-coated tissue culture dishes that were untreated, treated with active FAP, or treated with inhibited FAP. As shown in Fig 5A, both wild type and SR-A^-/-^ macrophages attached poorly to native type I collagen (buffer treated). Treating collagen with active FAP, but not with PMSF-inhibited FAP, significantly increased the attachment of wild-type, but not SR-A^-/-^ macrophages. In parallel, the majority of wild-type and SR-A^-/-^ macrophages remained rounded with a surface area < 100 μm^2^ when plated for 2 h on native type I collagen. In contrast, compared to SR-A^-/-^ macrophages, significantly more wild-type macrophages adopted a spread morphology with a surface area >100 μm^2^ when plated on type I collagen that was treated with active FAP, but not inactive FAP (Fig 5B). These results identify SR-A as the macrophage receptor responsible for increased adhesion to FAP-cleaved type I collagen.

### SR-A expressing TAMs localize to the tumor stroma

**Fig 6:**
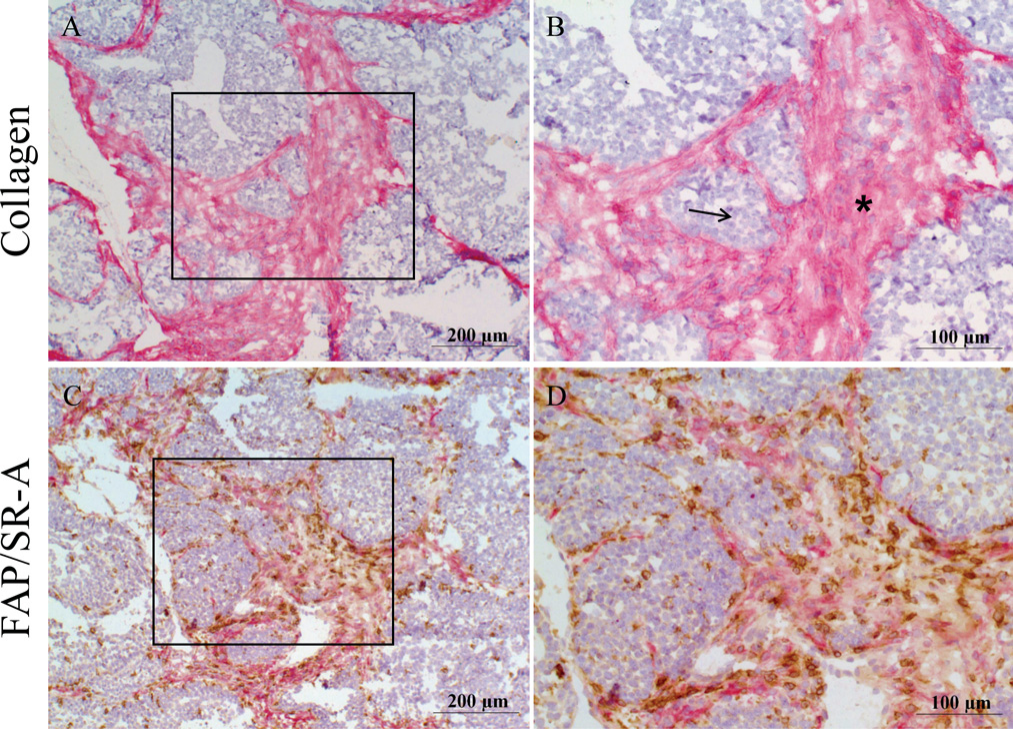
SR-A expressing macrophages localize in the tumor stroma. Consecutive frozen tumor sections were prepared from MMTV-PyMT mice and immunostained using A-B) a collagen antibody to detect stromal regions (red stain); C-D) a dual-stain technique with a FAP antibody to detect fibroblasts (red stain) and a SR-A antibody to visualize SR-A-positive macrophages (brown stain). Tissues were counterstained with hematoxylin and images digitally captured. B and D represent high power magnification (40x) of inset regions from A and C respectively. Arrows indicate acini of epithelial cells and asterisks indicate collagen-rich stroma.

The finding that FAP-cleaved collagen enhances SR-A-mediated macrophage adhesion suggests that SR-A-expressing TAMs should be enriched in areas of tumor where TAFs are abundant. To assess this prediction, we isolated mammary tumors from female MMTV-PyMT mice and performed immunostaining for collagen, and dual staining for FAP, SR-A that would indicate co-localization of TAFs and TAMs, respectively. As shown in Fig 6, the breast tumors are characterized by a distinct collagen-rich stromal compartment surrounding the acini of malignant epithelial cells. Importantly, dual staining for FAP-expressing TAFs and SR-A-expressing TAMs showed an abundant macrophage infiltrate into these tumors and that FAP-positive fibroblasts and SR-A-positive macrophages are localized to the same stromal areas in the tumors. (Fig 6). This co-localization of FAP-expressing TAFs and SR-A-expressing TAMs in the collagen-rich stroma supports the mechanism for localized stromal cell communication mediated by FAP and SR-A via the ECM modifications.

## Discussion

Our results demonstrate that FAP-mediated cleavage of collagen creates an adhesion substrate for macrophages that is recognized by SR-A. This conclusion is based on the increased attachment and spreading of macrophages on collagen treated with enzymatically active FAP, but not to collagen exposed to FAP that was enzymatically inactivated. The cation-independence of this adhesion and the disruption of this adhesive interaction by polyinosine, a compound that disrupts scavenger receptor interactions, suggested that SR-A mediates macrophage attachment to FAP-cleaved collagen. The specific importance of SR-A in mediating macrophage adhesion to FAP-cleaved collagen was confirmed by comparing the adhesion of wild type macrophages to those that lack SR-A. Together, these results identify FAP-cleaved type I collagen as a ligand for SR-A-mediated macrophage adhesion.

FAP is an activated fibroblast-specific protease that is implicated in modifications of tumor stroma [12, 14, 32, 33] and correlated with higher histological grades of malignancies, metastasis, and poor patient prognosis [34–36]. Importantly, FAP is not normally found in adult healthy tissues but occurs at sites of inflammation [12, 33, 37, 38]. Although the biological significance of specific FAP cleavage products is not known, there is increasing evidence that FAP activity plays an important role in modifying collagen matrices, particularly in tumors and atherosclerosis [13, 32, 39]. For example, the expression and proteolytic activity of FAP was correlated with the presence of macrophages in human atheroma [13]. In mouse tumor models, FAP^-/-^ and FAP enzymatic inhibition slowed tumor growth, increased stromal collagen content, and decreased collagen fibril organization in the tumor stroma compared to tumor tissues isolated from FAP^+/+^ mice [32]. It was further shown that FAP-targeted elimination of TAFs in the 4T1 mouse model of breast cancer reduced infiltration of TAMs and other immune cells to the tumor site [40]. Our results demonstrating that FAP-cleaved collagen increases macrophage adhesion suggest a novel mechanism by which FAP regulates inflammatory responses within tumors by modulating the behavior of macrophages.

Macrophages are key contributors to many chronic inflammatory conditions [41–44]. Targeted influx and accumulation of macrophages are important determinants of macrophage function, and critical to the regulation of inflammatory, immune, and repair processes [45–47]. In cancer, macrophage accumulation and interaction with other cells in the tumor microenvironment are linked to poor patient prognosis [15–18]. Thus, an important implication of our findings is the possibility that increased FAP expression by TAFs in the tumor stroma results in the cleavage of type I collagen to promote the localized accumulation of macrophages.

A key feature of the model described above is that the adhesion of macrophages in tumors and/or chronic inflammatory sites is modulated by ECM modification. Macrophages express multiple receptors that recognize ECM components, including integrins and pattern recognition receptors (PRRs) such as SR-A [48–50]. SR-A mediates the cation-independent adhesion of macrophages to several modified ECM components including heat-denatured collagen and collagenase-treated type I collagen, but does not bind to native collagen [20, 24, 25, 51]. Our findings suggest that a novel biological function of FAP proteolysis of type I collagen is to expose previously masked adhesion sites for SR-A on the collagen. Although MMPs such as collagenase are known to aggressively degrade and convert native type I collagen into an adhesive substrate for SR-A, FAP is a serine protease that cleaves collagen differently than MMPs creating products that are not the same as those generated by collagenases [52–54]. Thus, one cannot assume a priori that previous findings using collagenase to degrade collagen [e.g., [20]] apply to FAP-cleaved collagen. Nonetheless, our results showing that macrophage adhesion to FAP-cleaved collagen is cation-independent, inhibited by polyinosine, and absent in SR-A^-/-^ macrophages identify SR-A as a macrophage receptor that mediates adhesion to FAP-cleaved collagen.

The role of SR-A in infection, atherosclerosis and Alzheimer’s disease are well studied [reviewed in [55, 56]]. However, little is known about the contributions of SR-A to the formation and progression of tumors. Recent reports suggest that SR-A may be a marker for macrophages present in aggressive tumors [57, 58]. Importantly, Neyen et al. demonstrated that SR-A deficiency in vivo protects mice from tumor progression and metastasis [59]. In addition, we previously reported that SR-A-mediated adhesion activates multiple signaling cascades including Lyn-PI3K and PLA_2_-12/15LOX pathways [22, 23]. We further showed that SR-A-dependent macrophage adhesion induces the release of PGE_2_ which acts in an autocrine/paracrine mechanism to inhibit TNFα production and increase secretion of IL-10 [60]. Other studies examining the role of SR-A in mouse models of inflammatory disease demonstrated a similar association of SR-A with a tumor supportive M2 macrophage phenotype [61–63]. These reports suggest that in addition to promoting macrophage retention, SR-A-mediated interaction with components of the tumor microenvironment may contribute to the regulation of TAM phenotype.

### Conclusion

Our results show that FAP cleavage of type I collagen promotes macrophage adhesion (attachment and spreading), and that these interactions are specifically mediated by macrophage class A scavenger receptors (SR-A/CD204). We further show that FAP positive TAFs and SR-A positive TAMs co-localize in the stroma of breast tumors from MMTV-PyMT mice. Together our results implicate a novel biological role for FAP-cleaved type I collagen, and define a mechanism for dialog between fibroblasts and macrophages via a FAP-ECM-SR-A axis, that could increase macrophage retention and function in tumors and potentially other inflammatory sites.
